# Deregulation of hsa_circ_0001971/miR-186 and hsa_circ_0001874/miR-296 signaling pathways promotes the proliferation of oral squamous carcinoma cells by synergistically activating SHP2/PLK1 signals

**DOI:** 10.1038/s41598-021-99488-2

**Published:** 2021-10-18

**Authors:** Wang Jun, Ouyang Shaobo, Zhang Xianhua, Zhao Siyu, Cheng Mingyang, Fan Xin, Cai Ying, Liao Lan

**Affiliations:** 1 Oral and Maxillofacial Surgery, Second Affiliated Hospital of Nanchang University, Nanchang, 330006 China; 2 Department of Oral Prosthodontics, Affiliated Stomatological Hospital of Nanchang University, Jiangxi Provincial Key Laboratory of Oral Biomedicine, 49 Fuzhou Lu, Nanchang, 330006 China

**Keywords:** Cell biology, Molecular biology

## Abstract

It has been demonstrated that circ_0001874 and circ_0001971 are potential biomarkers for the diagnosis of oral squamous carcinoma (OSCC). MiR-186 was reported to serve as a tumor suppressor in OSCC, and the down-regulation of miR-186 was reported to lead to higher expression of oncogenic factor SHP2 and the activation of growth promoting signaling. In this study, we aimed to explore the possible molecular role of circ_0001874 and circ_0001971 signaling in the pathogenesis of OSCC. RT-qPCR, Western blot, online bioinformatics tools and luciferase assay were utilized to study the molecular signaling pathways of circ_0001874 and circ_0001971. MTT assay and FCM assay were performed to investigate the synergistic effect of circ_0001971 and circ_0001874 on cell proliferation and apoptosis. By observing the effect of different miRNAs on the levels of circ_0001847 and circ_0001971, it was identified that circ_0001847 and circ_0001971 respectively sponged the expression of miR-296 and miR-186 via binding to these miRNAs. Also, SHP2 mRNA and PLK1 mRNA were respectively targeted by miR-186 and miR-296-5p. We also established two signaling pathways, i.e., circ_0001971/miR-186/SHP2 and circ_0001874/miR-296-5p/PLK1, and validated the synergistic effect of circ_0001971 and circ_0001874 via observing their positive effect on cell proliferation and negative effect on cell apoptosis. The expression of miR-186 and miR-296-5p was generally lower in saliva of OSCC patients compared with that in OLK patients, while the expression of miR-186 and miR-296-5p was specifically up-regulated in saliva of OSCC patients. In conclusion, the finding of this study demonstrated that the relative level of hsa_circ_0001971 and hsa_circ_0001874 were different in the saliva of OSCC patients and could be used as predictive biomarkers for the development of OSCC. Furthermore, oncogenic effects of hsa_circ_0001971 and hsa_circ_0001874 in the development of OSCC might be, at least partially, mediated by its downstream signaling pathways including hsa_circ_0001971/microRNA-186/SHP2 and hsa_circ_0001874/microRNA-297/PLK1.

## Introduction

Oral squamous cell carcinoma (OSCC) ranks the ninth among all malignant tumors worldwide^1^. In spite of the advances in the development of surgical procedures, many OSCC patients have been diagnosed with advanced OSCC with poor prognosis, i.e., < 60% of 5-year survival^2^. Traditional tumor markers including serum antigen of squamous cell carcinoma, carcinoembryonic antigen and carbohydrate antigen have all been used in the diagnosis of OSCC patients 19–9. Nevertheless, because of the insufficient sensitivity and specificity in the diagnosis of OSCC, most OSCC patients cannot be diagnosed in their early stage^3^. For that reason, new biomarkers of high specificity and sensitivity are needed for early OSCC diagnosis.

Circular RNAs (circRNAs) belong to a family of endogenous RNAs featured by the covalently closed loop structure containing no 3′ poly (A) tail or 5′ cap^4^. Increasing evidence has shown the important roles played by circRNAs in regulating gene expression at the post-transcriptional level^5^. With the better understanding on the role of circRNAs, it was revealed that circRNAs are involved in the onset of numerous types of illnesses, such as cardiovascular diseases, cancer, and neurological disorders^6–8^. Our previous study demonstrated that hsa_circ_0001874 and hsa_circ_0001971 could be potentially used as biomarkers for the diagnosis of OSCC^9^. It has been illustrated that circRNAs work as completing endogenous RNAs (ceRNAs) to suppress the function and activity of target miRNAs^5,10,11^.

It was demonstrated that the increased expression of miR-296-5p in K1 cells inhibited the expression of PLK1. The miR-296-5p transfection in K1 cells also arrested the cells in the G2/M phase to block cell proliferation and colony formation. By targeting PLK1 expression, the presence of miR-296-5p can block the growth of PTC cells while promoting their apoptosis^12^. The expression of PLK1 was associated with the progression of tumorigenesis and the poor prognosis in OSCC patients. Furthermore, the expression of PLK1 is much greater in tumor tissues of lung cancer, cholangiocarcinoma, head and back squamous cell carcinoma, and uterine corpus endometrial carcinoma than that in normal tissues^13,14^.

The results obtained from both TargetScan and miRanda indicated the role of SHP2 as a miR-186 target. More significantly, a negative correlation between SHP2 and miR-186 was demonstrated. It was also revealed that SHP2 acts as a direct miR-186 target in regulating EGFR signaling^15^. In OSCC, SHP2 was discovered to become overexpressed in cancer cells, but the inhibition of SHP2 decreased the rate of cell proliferation^16^. Furthermore, both the luciferase assay and Western blotting confirmed the role of SHP2 as a miR-186 target. Based on a previous research, it was additionally noted that the expression of SHP2 was increased in OSCC cells^16^. Furthermore, the downregulated miR-186 expression resulted in greater SHP2 expression and the activation of growth promoting signals. Thereby, miR-186 might be used as a novel and effective agent in OSCC therapy^15^. Recently, Shp2 was discovered to be involved in Plk1 activation and the maintenance in the stability of chromosomes^17^. More mechanistic studies have shown that the GOF mutant of Shp2 hyperactivates Plk1 by promoting its phosphorylation^18^.

Considering that hsa_circ_0001874 and hsa_circ_0001971 could be potentially used as biomarkers for the diagnosis of OSCC^9^, and miR-296 and miR-186 could interact with hsa_circ_0001874 and hsa_circ_0001971 respectively (circinteractome.nia.nih.gov), we hypothesized that upregulation of hsa_circ_0001874 and hsa_circ_0001971 may functionally contribute to the tumorigenesis of OSCC via modulating the expression of miR-296 and miR-186, as well as their downstream effectors such as SHP2 and PLK1. In this study, we aimed to explore the possible molecular mechanism underlying the tumorigenic effect of hsa_circ_0001874 and hsa_circ_0001971 in the pathogenesis of OSCC.

## Materials and methods

### Patient recruitment

In this study, several patient cohorts were studied. First, we recruited two group of patients, i.e., a total of 135 OSCC patients and a total of 105 oral leukoplakia (OLP) patients, who were accordingly divided into two groups, i.e., an OSCC group (N = 135) and an OLK group (N = 105). The OSCC patients subsequently underwent relevant operations and their saliva samples were collected. All participants were then divided into two groups, i.e., 1: Group of pre-operation OSCC patients (N = 135); and 2. Group of post-operation OSCC patients (N = 135). The Human Research Ethics Committees of Affiliated Stomatological Hospital of Nanchang University has approved this research and all methods were performed in accordance with the last vision of the Declaration of Helsinki. Written informed consent was obtained from all patients or their first-degree relatives before the study.

### Cell culture and transfection

SCC-9 cells were directly purchased from American Type Culture Collection (Manassas, VA). According to the protocol of this study, the SCC-9 cells were cultured in a DMEM (Thermo Fisher Scientific, Grand Isle, NY) added with sodium pyruvate, L-glutamine, streptomycin, penicillin, β-mercaptoethanol (Sigma, St. Louis, MO), and 10% endotoxin-free FBS (Gemi, Sacramento, CA). After the SCC-9 cells reached logarithmic growth, they were used to establish different cell models as shown below.

In cell treatment group 1, the SCC-9 cells were divided into 17 groups, i.e., 1. NC group (SCC-9 cells transfected with a negative control); 2. pre-miR-146b group (SCC-9 cells transfected with 30 nM and 60 nM pre-miR-146b respectively); 3. pre-miR-149 group (SCC-9 cells transfected with 30 nM and 60 nM pre-miR-149 respectively); 4. pre-miR-515 group (SCC-9 cells transfected with 30 nM and 60 nM pre-miR-515 respectively); 5. pre-miR-532 group (SCC-9 cells transfected with 30 nM and 60 nM pre-miR-532 respectively); 6. pre-miR-585 group (SCC-9 cells transfected with 30 nM and 60 nM pre-miR-585 respectively); 7. pre-miR-87 group (SCC-9 cells transfected with 30 nM and 60 nM pre-miR-87 respectively); 8. pre-miR-940 group (SCC-9 cells transfected with 30 nM and 60 nM pre-miR-940 respectively); 9. pre-miR-127 group (SCC-9 cells transfected with 30 nM and 60 nM pre-miR-127 respectively); 10. pre-miR-194 group (SCC-9 cells transfected with 30 nM and 60 nM pre-miR-194 respectively); 11. pre-miR-324 group (SCC-9 cells transfected with 30 nM and 60 nM pre-miR-324 respectively); 12. pre-miR-662 group (SCC-9 cells transfected with 30 nM and 60 nM miR-662 respectively); 13. pre-miR-296 group (SCC-9 cells transfected with 30 nM and 60 nM pre-miR-296 respectively); 14. pre-miR-661 group (SCC-9 cells transfected with 30 nM and 60 nM pre-miR-661 respectively); 15. pre-miR-662 group (SCC-9 cells transfected with 30 nM and 60 nM pre-miR-662 respectively); 16. pre-miR-186 group (SCC-9 cells transfected with 30 nM and 60 nM pre-miR-186 respectively); and 17. pre-miR-607 group (SCC-9 cells transfected with 30 nM and 60 nM pre-miR-607 respectively).

In cell treatment group 2, the SCC-9 cells were divided into 5 groups, i.e., 1. Empty vector group (SCC-9 cells transfected with an empty vector); 2. hsa_circ_0001874 group (SCC-9 cells transfected with vectors carrying hsa_circ_0001874); 3. hsa_circ_0001971 group (SCC-9 cells transfected with vectors carrying hsa_circ_0001971); 4. pre-miR-186 group (SCC-9 cells transfected with pre-miR-186); and 5. pre-miR-296 group (SCC-9 cells transfected with pre-miR-296). Since miRNA precursors were used, a scramble control group (SCC-9 cells transfected with scramble controls), an empty vector + scramble control group (SCC-9 cells transfected with an empty vector and scramble controls), an hsa_circ_0001874 + scramble control group (SCC-9 cells transfected with vectors carrying hsa_circ_0001874 and scramble controls) and an hsa_circ_0001971 + scramble control group (SCC-9 cells transfected with vectors carrying hsa_circ_0001971 and scramble controls) were also established. However, since the results were similar among the scramble control groups and the non-scramble control groups, we did not present the data of scramble control groups.

In cell treatment group 3, the SCC-9 cells were divided into 3 groups, i.e., 1. NC siRNA group (SCC-9 cells transfected with a negative control siRNA); 2. hsa_circ_0001874 siRNA group (SCC-9 cells transfected with hsa_circ_0001874 siRNA); and 3. hsa_circ_0001971 siRNA group (SCC-9 cells transfected with hsa_circ_0001971 siRNA).

In cell treatment group 4, the SCC-9 cells were divided into 3 groups, i.e., 1. NC siRNA group (SCC-9 cells transfected with a negative control siRNA); 2. hsa_circ_0001971 siRNA (50 nM) group (SCC-9 cells transfected with 50 nM of hsa_circ_0001971 siRNA); and 3. hsa_circ_0001971 (100 nM) group (SCC-9 cells transfected with 100 nM of hsa_circ_0001971 siRNA).

In cell treatment group 5, the SCC-9 cells were divided into 3 groups, i.e., 1. NC siRNA group (SCC-9 cells transfected with a negative control siRNA); 2. hsa_circ_0001874 siRNA (50 nM) group (SCC-9 cells transfected with 50 nM of hsa_circ_0001874 siRNA); and 3. hsa_circ_0001874 siRNA (100 nM) group (SCC-9 cells transfected with 100 nM of hsa_circ_0001874 siRNA).

In cell treatment group 6, the SCC-9 cells were divided into 7 groups, i.e., 1. Empty vector group (SCC-9 cells transfected with an empty vector); 2. hsa_circ_0001874 group (SCC-9 cells transfected with vectors carrying hsa_circ_0001874); 3. hsa_circ_0001971 group (SCC-9 cells transfected with vectors carrying hsa_circ_0001971); 4. hsa_circ_0001874 + hsa_circ_0001971 group (SCC-9 cells transfected with vectors carrying hsa_circ_0001874 and vectors carrying hsa_circ_0001971); 5. hsa_circ_0001874 + hsa_circ_0001971 + pre-miR-186 (SCC-9 cells transfected with vectors carrying hsa_circ_0001874 and vectors carrying hsa_circ_0001971 along with pre-miR-186); 6. hsa_circ_0001874 + hsa_circ_0001971 + pre-miR-296 (SCC-9 cells transfected with vectors carrying hsa_circ_0001874 and vectors carrying hsa_circ_0001971 along with pre-miR-296); and 7. hsa_circ_0001874 + hsa_circ_0001971 + pre-miR-186 + pre-miR-296 group (SCC-9 cells transfected with vectors carrying hsa_circ_0001874 and vectors carrying hsa_circ_0001971 along with pre-miR-186 and pre-miR-296).

In all cell groups, the cells were transfected with Lipofectamine 2000 (Invitrogen, Carlsbad, CA) following the standard operating protocol provided by the manufacturer. And all the precursors and siRNAs were manufactures and produced by Sigma-Aldrich, St. Louis, MO, US.

### RNA isolation and real-time PCR

Real-time PCR was used to assay the relative expression of miR-146b-3p, miR-149, miR-515-3p, miR-532-3p, miR-585, miR-874, miR-940, miR-127-5p, miR-194, miR-324-5p, miR-662, miR-296-5p, miR-661, miR-662, miR-186 miR-607, circ_0001874, circ_0001971, SHP2 mRNA, and PLK1 mRNA in each sample. In brief, the samples were first homogenated in 200 μl of chloroform and centrifuged for 15 min at 12,000 × g and 4 °C to collect the supernatant, which was subject to a phenol-based procedure (Trizol LS assay kit, Invitrogen, Carlsbad, CA) or a column-based procedure (miRNeasy, Thermo Fisher Scientific, CA). In the next step, the extract total RNA was subject to reverse transcription with a PrimeScript RT assay kit (TaKaRa, Tokyo, Japan). The mature miRNAs were reverse transcribed with a TaqMan™ MicroRNA Reverse Transcription Kit (Thermo Fisher Scientific, CA). Then, real time PCR was carried out by utilizing a 7500c real-time PCR machine (ABI, Foster City, CA) in conjunction with a SYBR Premix EX Taqman assay kit (TaKaRa, Tokyo, Japan). Finally, the relative expression of miR-146b-3p, miR-149, miR-515-3p, miR-532-3p, miR-585, miR-874, miR-940, miR-127-5p, miR-194, miR-324-5p, miR-662, miR-296-5p, miR-661, miR-662, miR-186 miR-607, circ_0001874, circ_0001971, SHP2 mRNA, and PLK1 mRNA in each sample was determined using the Ct values. The primers used in this experiment were obtained from OriGene Technologies, Rockville, MD, US. The specificity of circ_0001874 and circ_0001971 was determined by electrophoresis, melting curve analysis, and sequencing. The back-splice junction sequences of circ_0001874 and circ_0001971 were confirmed by Sanger sequencing, which indicated results consistent with the database (http://www.circbase.org/cgi-bin/simplesearch.cgi).

### Luciferase assay

Luciferase assays were carried out in this study to verify the regulatory relationships of miR-146b-3p/circ_0001874, miR-149/circ_0001874, miR-296-5p/circ_0001874, miR-515-3p/circ_0001874, miR-532-3p/circ_0001874, miR-585/circ_0001874, miR-661/circ_0001874, miR-662/circ_0001874, miR-874/circ_0001874, miR-940/circ_0001874, miR-127-5p/circ_0001971, miR-186/circ_0001971, miR-194/circ_0001971, miR-324-5p/circ_0001971, miR-607/circ_0001971, miR-626/circ_0001971, miR-186/SHP2 mRNA, and miR-296/PLK1 mRNA, respectively. In brief, the gene sequences of circRNAs or mRNAs carrying the binding sites of their respective target miRNAs were cloned into pcDNA3.1 vectors (Promega, Madison, WI) to generate wild type vectors. In the next step, a site-directed mutagenesis assay kit (Stratagene, San Diego, CA) was used following the standard operating protocol provided by the manufacturer to generate mutant sequences of the binding sites of candidate miRNAs, which were also cloned into pcDNA3.1 vectors to generate mutant type vectors. Then, SCC-9 cells were co-transfected with mutant type/wild type vectors of circRNAs or mRNAs along with the mimicry of their target miRNAs at 50 nM and 100 nM (OriGene Technologies, Rockville, MD, US). The luciferase activity of transfected cells was analyzed using a Dual Luciferase Reporter assay kit (Promega, Madison, WI) at 48 h post transfection on a TD-20/20 luminometer (Turner Designs, Sunnyvale, CA).

### MTT assay

The proliferation of treated cells was analyzed by using an MTT assay (Dojindo Molecular, Kumamoto, Japan) following the standard operating protocol provided by the manufacturer. The optical density of every well was measured at a 450 nm wavelength on a microplate reader (Bio-Rad Laboratories, Hercules, CA).

### FCM assay

The apoptosis of treated cells was analyzed by utilizing an Annexin V-fluorescein isothiocyanate (FITC)/propidium iodide (PI) apoptosis assay kit (cat no. V13242; Thermo Fisher Scientific, Waltham, MA).

### Statistical analysis

All results were shown as mean ± standard deviations. Statistical comparisons between different groups were done by using Student’s t tests. All statistical analysis was done by SPSS 17.0 (SPSS, Chicago, IL). P-values of < 0.05 were considered statistically significant.

## Results

### Candidate miRNAs inhibited the expression of circRNAs

**Figure 1:**
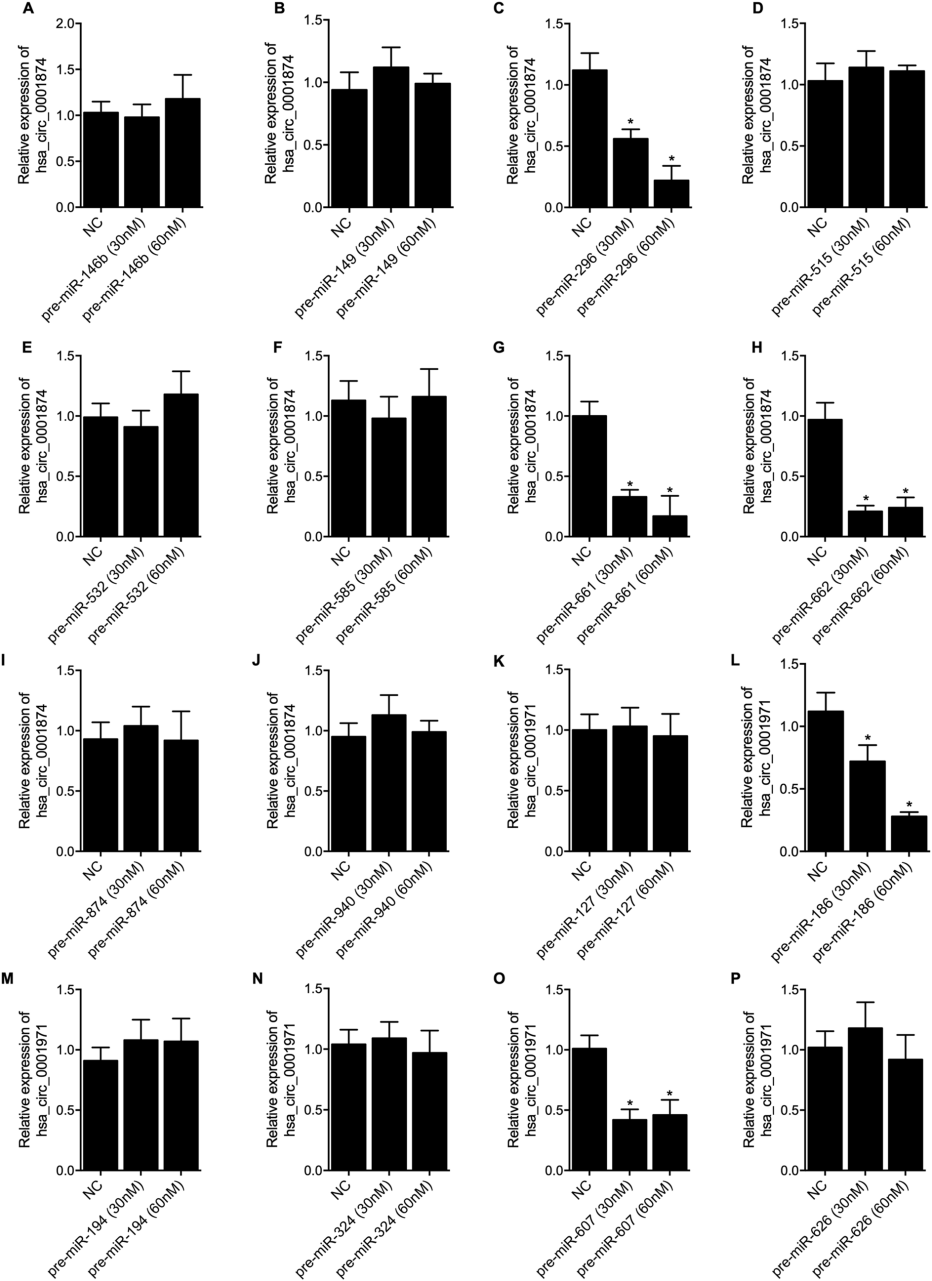
The potential regulatory relationships between candidate target miRNAs and circ_0001874 and circ_0001971. (**A**) The expression of circ_0001874 was comparable between the NC group and the miR-146b-3p precursor group; (**B**) The expression of circ_0001874 was comparable between the NC group and the miR-149 precursor group; (**C**) The expression of circ_0001874 was evidently decreased in the miR-296-5p precursor group than that in the NC group (*P value < 0.05 vs. NC group); (**D**) The expression of circ_0001874 was comparable between the NC group and the miR-515-3p precursor group; (**E**) The expression of circ_0001874 was comparable between the NC group and the miR-532-3p precursor group; (**F**) The expression of circ_0001874 was comparable between the NC group and the miR-585 precursor group; (**G**) The expression of circ_0001874 was evidently decreased in the miR-661 precursor group than that in the NC group (*P value < 0.05 vs. NC group); (**H**) The expression of circ_0001874 was evidently decreased in the miR-662 precursor group than that in the NC group (*P value < 0.05 vs. NC group); (**I**) The expression of circ_0001874 was comparable between the NC group and the miR-532-3p precursor group; (**J**) The expression of circ_0001874 was comparable between the NC group and the miR-940 precursor group; (**K**) The expression of circ_0001971 was comparable between the NC group and the miR-127-5p precursor group; (**L**) The expression of circ_0001971 was evidently decreased in the miR-186 precursor group than that in the NC group (*P value < 0.05 vs. NC group); (**M**) The expression of circ_0001971 was comparable between the NC group and the miR-532-3p precursor group; (**N**) The expression of circ_0001971 was comparable between the NC group and the miR-324-5p precursor group; (**O**) The expression of circ_0001971 was evidently decreased in the miR-607 precursor group than that in the NC group (*P value < 0.05 vs. NC group); (**P**) The expression of circ_0001971 was comparable between the NC group and the miR-662 precursor group.

Online bioinformatics tools (https://circinteractome.nia.nih.gov/index.html) were used to predict the target miRNAs of circ_0001874 and circ_0001971. And we selected candidate miRNAs for further functional analysis based on the predicted target genes of the miRNAs, i.e. only those miRNAs with possible target gene(s) involved in the tumorigenesis of OSCC were selected. The identified candidate miRNA precursors at different dosages (30 nM and 60 nM) were transfected into SCC-9 cells to observe the regulatory relationships between candidate miRNAs and circRNAs. As indicated by the RT-qPCR results, the over-expression of 11 candidate miRNAs at both 30 nM and 60 nM including miR-146b-3p (Fig. 1A), miR-149 (Fig. 1B), miR-515-3p (Fig. 1D), miR-532-3p (Fig. 1E), miR-585 (Fig. 1F), miR-874 (Fig. 1I), miR-940 (Fig. 1J), miR-127-5p (Fig. 1K), miR-194 (Fig. 1M), miR-324-5p (Fig. 1N) and miR-662 (Fig. 1P) had no influence on the expression of circRNAs. However, the transfection of miR-296-5p precursors at 30 nM (Fig. 1C), miR-661 precursors at 30 nM (Fig. 1G) and miR-662 precursors at 30 nM (Fig. 1H) markedly down-regulated the level of circ_0001874, and the transfection of miR-186 precursors at 30 nM (Fig. 1L) and miR-607 precursors at 30 nM (Fig. 1O) markedly down-regulated the level of circ_0001971. Meanwhile, the higher dosage of 60 nM of miR-296-5p precursors (Fig. 1C) more significantly down-regulated the level of circ_0001874, and the higher dosage of 60 nM of miR-186 precursors (Fig. 1L) more significantly down-regulated the level of circ_0001971.

### MiR-296-5p bound to circ_0001847 while miR-186 bound to circ_0001971

**Figure 2:**
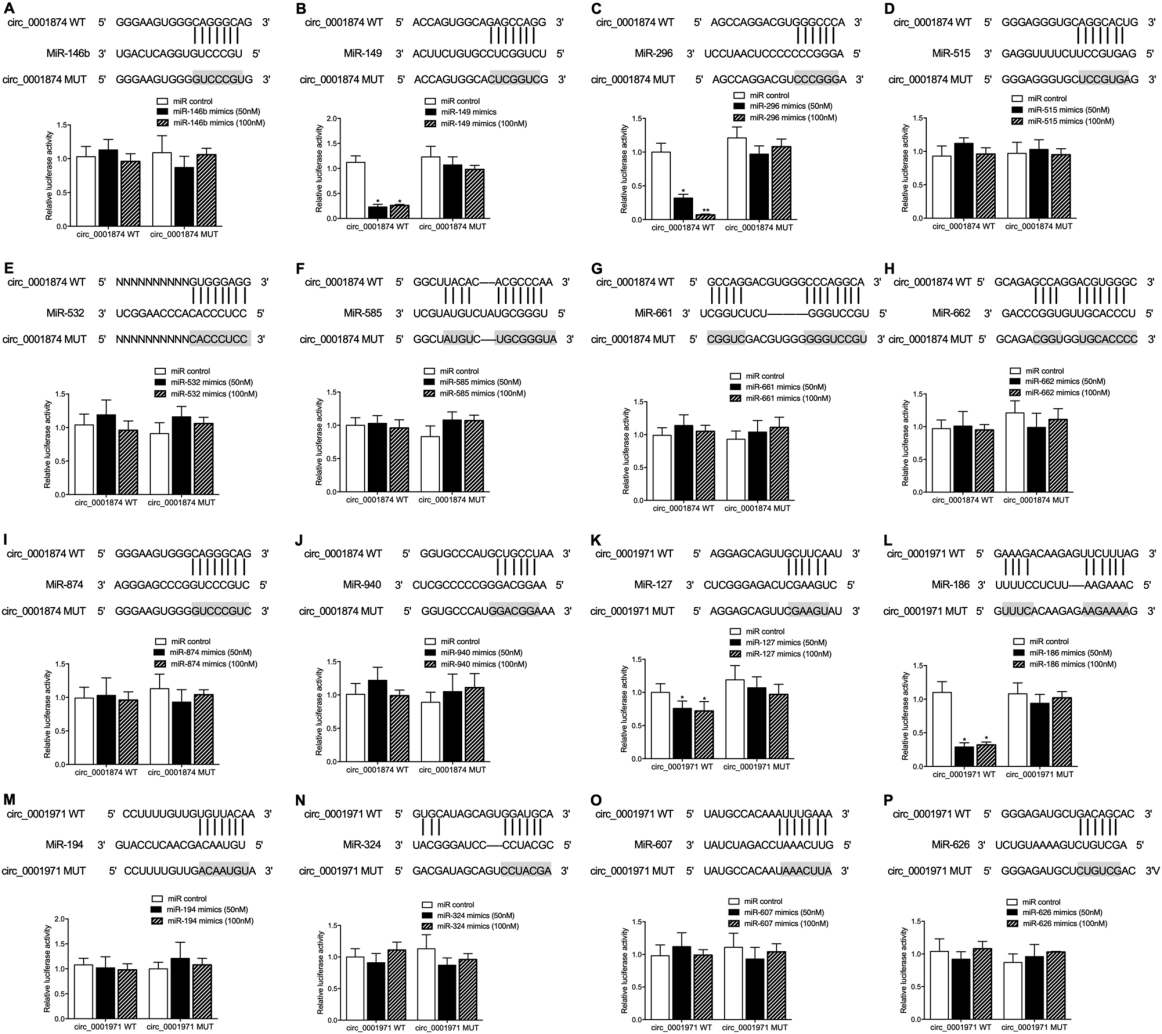
Computational analysis and luciferase assays validated the molecular relationships between miR-296-5p and circ_0001874, and between miR-186 and circ_0001971. (**A**) Computational analysis and luciferase assay of circ_0001847 and miR-146b-3p indicated no molecular relationship between circ_0001847 and miR-146b-3p; (**B**) Computational analysis and luciferase assay of circ_0001847 and miR-149 indicated that the luciferase activity of circ_0001847 was significantly reduced by miR-149 mimics at both high and low dosages (*P value < 0.05 vs. miR control); (**C**) Computational analysis and luciferase assay of circ_0001847 and miR-296 indicated that the luciferase activity of circ_0001847 was significantly reduced by miR-296 mimics in a dose-dependent manner (*P value < 0.05 vs. miR control); (**D**) Computational analysis and luciferase assay of circ_0001847 and miR-515-3p indicated no molecular relationship between circ_0001847 and miR-515-3p; (**E**) Computational analysis and luciferase assay of circ_0001847 and miR-532-3p indicated no molecular relationship between circ_0001847 and miR-532-3p; (**F**) Computational analysis and luciferase assay of circ_0001847 and miR-585 indicated no molecular relationship between circ_0001847 and miR-585; (**G**) Computational analysis and luciferase assay of circ_0001847 and miR-661 indicated no molecular relationship between circ_0001847 and miR-661; (**H**) Computational analysis and luciferase assay of circ_0001847 and miR-662 indicated no molecular relationship between circ_0001847 and miR-662; (**I**) Computational analysis and luciferase assay of circ_0001847 and miR-874 indicated no molecular relationship between circ_0001847 and miR-874; (**J**) Computational analysis and luciferase assay of circ_0001847 and miR-940 indicated no molecular relationship between circ_0001847 and miR-940; (**K**) Computational analysis and luciferase assay of circ_0001971 and miR-127 indicated that the luciferase activity of circ_0001971 was significantly reduced by miR-127 mimics at both high and low dosages (*P value < 0.05 vs. miR control); (**L**) Computational analysis and luciferase assay of circ_0001971 and miR-186 indicated that the luciferase activity of circ_0001971 was significantly reduced by miR-186 mimics at both high and low dosages (*P value < 0.05 vs. miR control); (**M**) Computational analysis and luciferase assay of circ_0001971 and miR-194 indicated no molecular relationship between circ_0001971 and miR-194; (**N**) Computational analysis and luciferase assay of circ_0001971 and miR-324 indicated no molecular relationship between circ_0001971 and miR-324-5p; (**O**) Computational analysis and luciferase assay of circ_0001971 and miR-607 indicated no molecular relationship between circ_0001971 and miR-607. (**P**) Computational analysis and luciferase assay of circ_0001971 and miR-662 indicated no molecular relationship between circ_0001971 and miR-662.

To further confirm the molecular interactions between miRNAs and circRNAs, luciferase assays were conducted (Fig. 2A-P). Accordingly, we found that the luciferase activity of wild-type hsa_circ_0001874 was inhibited by the transfection of miR-149 mimics (Fig. 2B) and miR-296 mimics (Fig. 2C), while no other mimics of miRNAs reduced the luciferase activity of wild-type circ_0001874. Also, only the transfection of miR-127 mimics (Fig. 2K) and miR-186 mimics (Fig. 2L) suppressed the luciferase activity of circ_0001971. Moreover, unlike miR-296 mimics which suppressed the luciferase activity of wild-type circ_0001874 at a dose-dependent manner, higher dosage of miR-149 mimics (100 nM), miR-127 mimics (100 nM) and miR-186 mimics (100 nM) exhibited similar inhibitory effect upon the luciferase activities. Furthermore, we tested the luciferase activity of wild-type hsa_circ_0001874 and hsa_circ_0001971 with according miRNA mimics at 200 nM, and we found that the inhibitory effect of 200 nM miRNA mimics was comparable with that 100 nM miRNA mimics (data not shown). Therefore, combined with the RT-qPCR results, it can be concluded that miR-296-5p and miR-186 respectively bind to circ_0001847 and circ_0001971 to down-regulate their expression.

### SHP2 mRNA and PLK1 mRNA were respectively targeted by miR-186 and miR-296-5p

**Figure 3:**
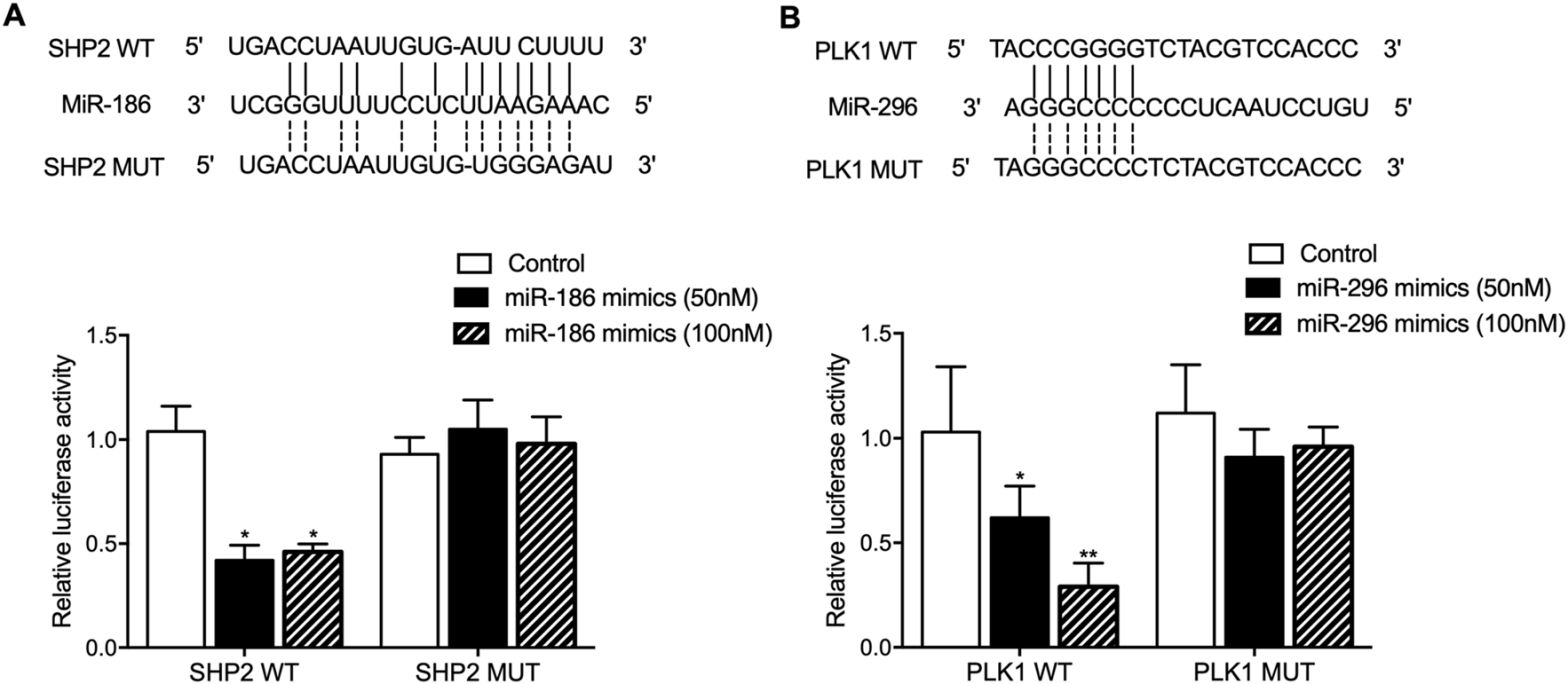
Computational analysis and luciferase assays validated the molecular relationships between miR-186 and SHP2, and between miR-296-5p and PLK1. (**A**) The putative binding site of miR-186 on the 3′UTR of SHP2 was identified, and the luciferase activity of wild-type SHP2 was significantly inhibited by the transfection of miR-186 mimics at both high and low dosages (*P value < 0.05 vs. control); (**B**) The putative binding site of miR-296-5p on the 3′UTR of PLK1 was identified, and the luciferase activity of wild-type PLK2 was significantly inhibited by the transfection of miR-296 mimics in a dose-dependent manner (*P value < 0.05 vs. control).

Although previous reports have validated the relationship between miR-186 and SHP2 in OSCC^11^ as well as the relationship between miR-296-5p and PLK1 in non-small cell lung cancer (NSCLC)^19^, we further performed computational analysis and luciferase assays to validate their molecular relationship. Accordingly, putative miR-186 (Fig. 3A) and miR-296-5p (Fig. 3B) binding sites were found on the 3′UTRs of SHP2 and PLK1, respectively, and the luciferase assays showed that the luciferase activity of wild-type SHP2 was inhibited by the transfection of miR-186 mimics (50 nM), while the higher dosage of miR-186 mimics (100 nM) exhibited similar inhibitory effect upon the luciferase activity of wild-type SHP2 (Fig. 3A). Also, the luciferase activity of wild-type PLK1 was inhibited by the transfection of miR-296 mimics (50 nM), while the higher dosage of miR-296 mimics (100 nM) more significantly reduced the luciferase activity of wild-type PLK1 (Fig. 3B). We also tested the luciferase activity of wild-type PLK1 with 200 nM miR-296 mimics, and we found that the inhibitory effect of 200 nM miR-296 mimics was comparable with that 100 nM miR-296 mimics (data not shown). Therefore, it can be concluded that SHP2 mRNA and PLK1 mRNA were respectively targeted by miR-186 and miR-296-5p.

### Establishment of the circ_0001874/circ_0001971 signaling

**Figure 4:**
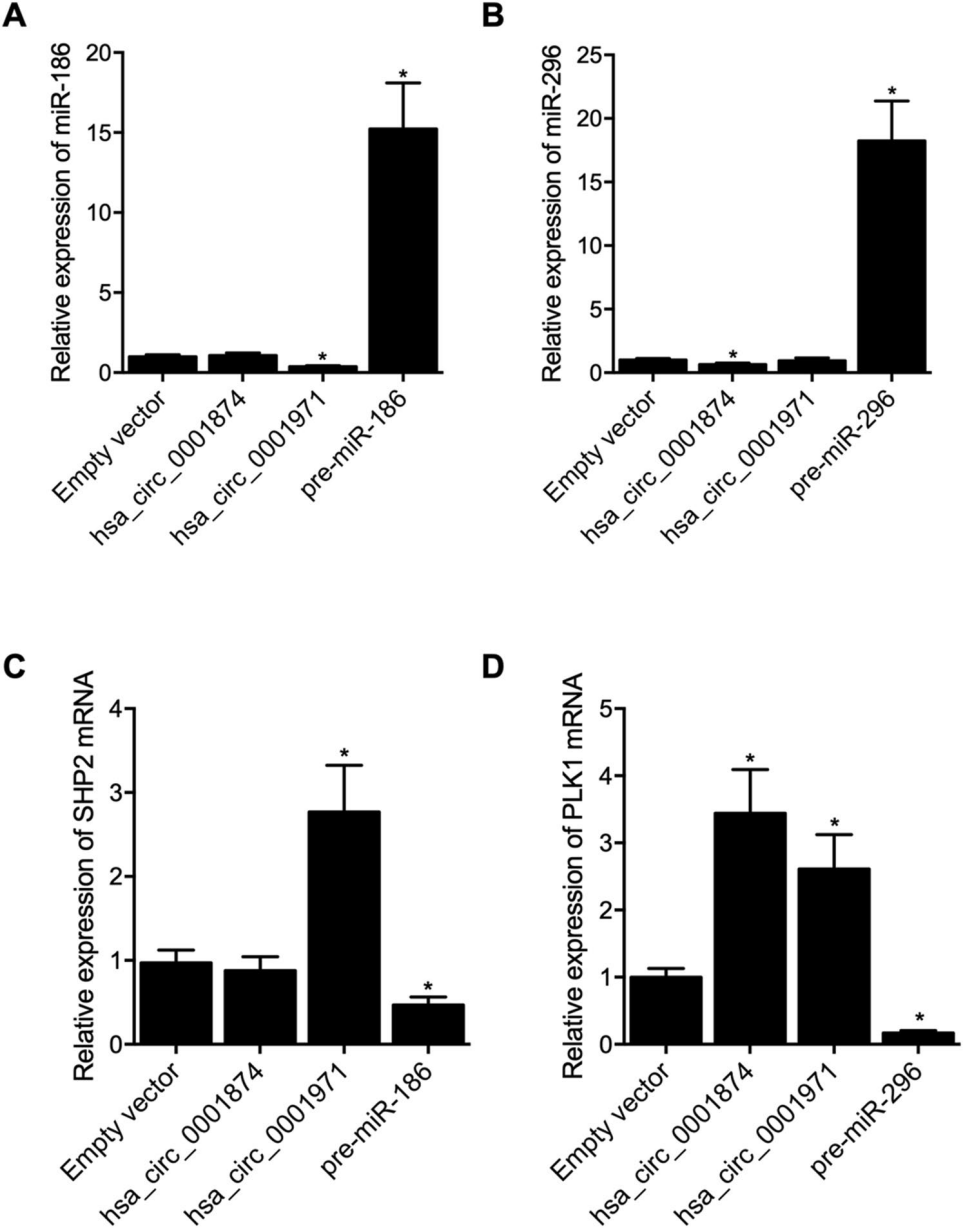
The overexpression of hsa_circ_0001874 and hsa_circ_0001971, as well the overexpression of miR-186 and miR-296, exhibited different effects upon the gene expression of PLK1 and SHP2. (**A**) Relative expression of miR-186 was evidently suppressed in the hsa_circ_0001971 group rather than in the hsa_circ_0001874 group. The successful transfection of miR-186 precursors was validated by evidently up-regulated miR-186 expression (*P value < 0.05 vs. empty vector group); (**B**) Relative expression of miR-296 was evidently suppressed in the hsa_circ_0001874 group rather than in the hsa_circ_0001971 group. The successful transfection of miR-296 precursors was validated by evidently up-regulated miR-296 expression (*P value < 0.05 vs. empty vector group); (**C**) Relative expression of SHP2 mRNA was significantly increased in the hsa_circ_0001971 group rather than in the hsa_circ_0001874 group. The overexpression of miR-186 significantly down-regulated the relative expression of SHP2 mRNA (*P value < 0.05 vs. empty vector group); (**D**) Relative expression of PLK1 mRNA was significantly higher in both hsa_circ_0001874 and hsa_circ_0001971 groups. The overexpression of miR-296 significantly down-regulated the relative expression of PLK1 mRNA (*P value < 0.05 vs. empty vector group).

SCC-9 cells were transfected with vectors carrying circ_0001874 or circ_0001971. Compared with other cell groups, miR-186 (Fig. 4A) and miR-296 (Fig. 4B) expression was evidently down-regulated in cells over-expressing circ_0001971 and circ_0001874, respectively. Meanwhile, the successful transfection of miR-186 precursors and miR-296 precursors was respectively validated by significantly up-regulated miR-186 expression (Fig. 4A) and miR-296 expression (Fig. 4B) in SCC-9 cells. Moreover, the expression of SHP2 mRNA (Fig. 4C) was significantly elevated by the over-expression of circ_0001971 but down-regulated by the over-expression of miR-186, and the expression of PLK1 mRNA (Fig. 4D) was significantly increased upon the transfection of circ_0001874 but evidently suppressed by the over-expression of miR-296.

**Figure 5:**
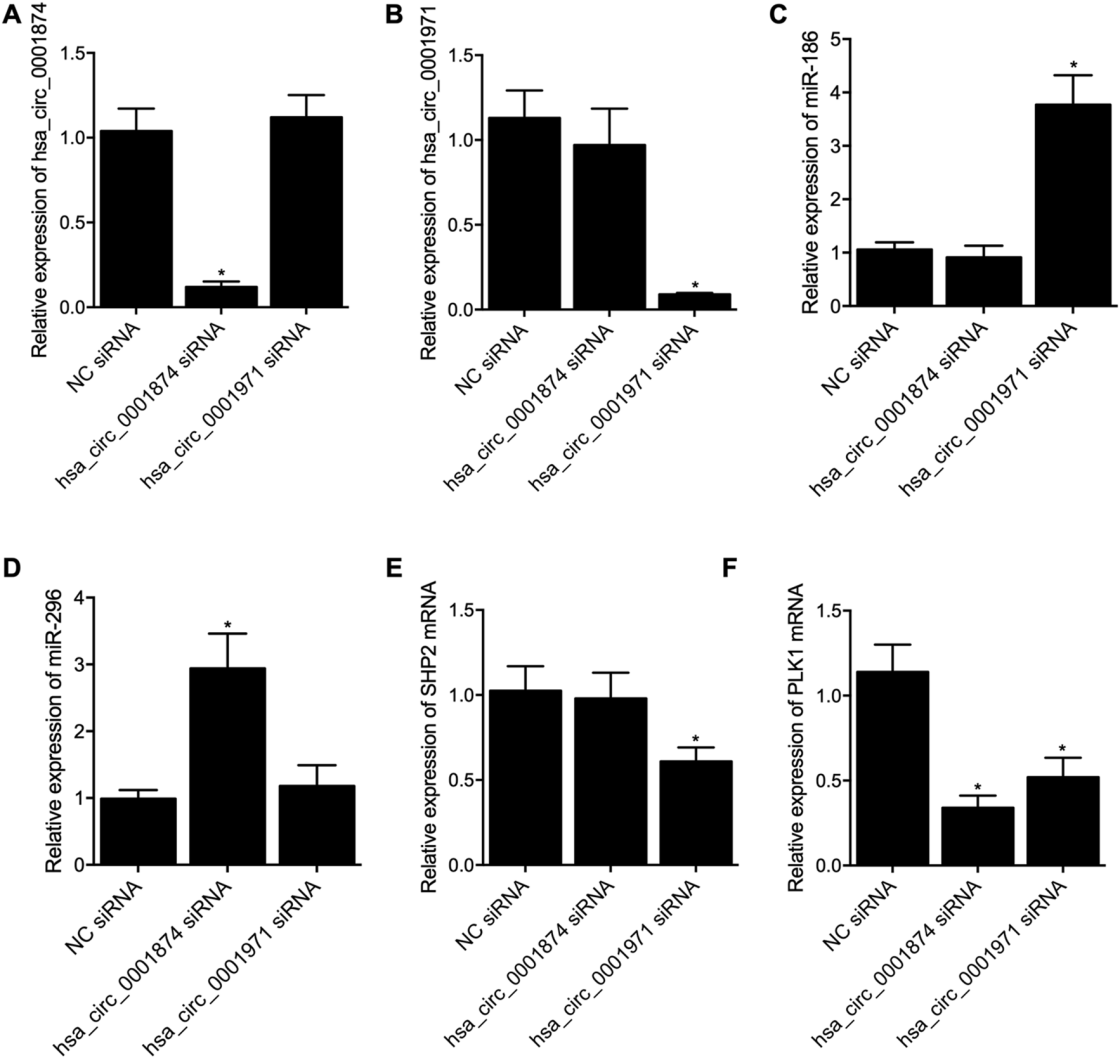
The knockdown of hsa_circ_0001874 and hsa_circ_0001971 up-regulated the gene expression of PLK1 and SHP2 via the regulation of miR-186 and miR-296 expression. (**A**) Relative expression of hsa_circ_0001874 was evidently suppressed by the transfection of hsa_circ_0001874 siRNA rather than the transfection of hsa_circ_0001971 siRNA (*P value < 0.05 vs. NC siRNA group); (**B**) Relative expression of hsa_circ_0001971 was evidently suppressed by the transfection of hsa_circ_0001971 siRNA rather than the transfection of hsa_circ_0001874 siRNA (*P value < 0.05 vs. NC siRNA group); (**C**) Relative expression of miR-186 was significantly elevated by the transfection of hsa_circ_0001971 siRNA rather than the transfection of hsa_circ_0001874 siRNA (*P value < 0.05 vs. NC siRNA group); (**D**) Relative expression of miR-296 was significantly elevated by the transfection of hsa_circ_0001874 siRNA rather than the transfection of hsa_circ_0001971 siRNA (*P value < 0.05 vs. NC siRNA group); (**E**) Relative expression of SHP2 mRNA was evidently inhibited in the hsa_circ_0001971 siRNA group rather than in the hsa_circ_0001874 siRNA group (*P value < 0.05 vs. NC siRNA group); (**F**) Relative expression of PLK1 mRNA was evidently inhibited in both the hsa_circ_0001874 siRNA and hsa_circ_0001971 siRNA groups (*P value < 0.05 vs. NC siRNA group).

Furthermore, SCC-9 cells were divided into 3 groups: 1. NC siRNA group; 2. Circ_0001874 siRNA group; 3. Circ_0001971 siRNA group. The expression of circ_0001874 (Fig. 5A) and PLK1 mRNA (Fig. 5F) was down-regulated by circ_0001874 siRNA. The expression of circ_0001971 (Fig. 5B) and SHP2 mRNA (Fig. 5E) was markedly inhibited by the transfection of circ_0001971 siRNA. The expression of miR-186 (Fig. 5C) and miR-296 (Fig. 5D) was respectively promoted by the transfection of circ_0001971 siRNA and circ_0001874 siRNA.

**Figure 6:**
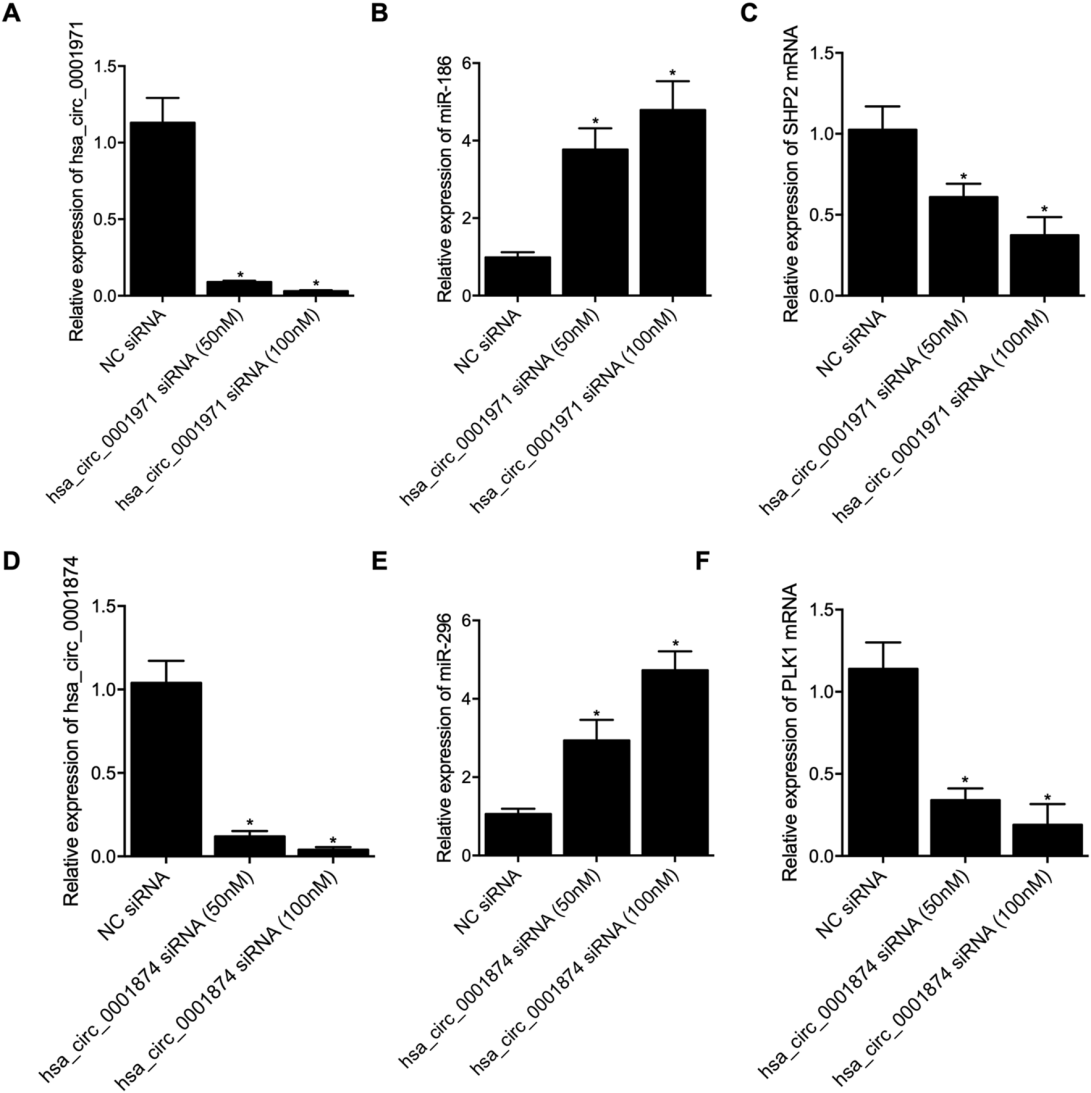
The knockdown of hsa_circ_0001874 and hsa_circ_0001971 up-regulated the gene expression of PLK1 and SHP2 via the regulation of miR-186 and miR-296 expression in a dose-dependent manner. (**A**) Relative expression of hsa_circ_0001971 was evidently suppressed by the transfection of hsa_circ_0001971 siRNA in a dose-dependent manner, as compared with that in the NC siRNA group (*P value < 0.05 vs. NC siRNA group); (**B**) Relative expression of miR-186 was significantly elevated by the transfection of hsa_circ_0001971 siRNA in a dose-dependent manner, as compared with that in the NC siRNA group (*P value < 0.05 vs. NC siRNA group); (**C**) Relative expression of SHP2 mRNA was evidently inhibited by the transfection of hsa_circ_0001971 siRNA group in a dose-dependent manner, as compared with that in the NC siRNA group (*P value < 0.05 vs. NC siRNA group); (**D**) Relative expression of hsa_circ_0001874 was evidently suppressed by the transfection of hsa_circ_0001874 siRNA in a dose-dependent manner, as compared with that in the NC siRNA group (*P value < 0.05 vs. NC siRNA group); (**E**) Relative expression of miR-296 was significantly elevated by the transfection of hsa_circ_0001874 siRNA in a dose-dependent manner, as compared with that in the NC siRNA group (*P value < 0.05 vs. NC siRNA group); (**F**) Relative expression of PLK1 mRNA was evidently inhibited by the transfection of hsa_circ_0001874 siRNA in a dose-dependent manner, as compared with that in the NC siRNA group (*P value < 0.05 vs. NC siRNA group).

Also, we tested the effect of circ_0001874 siRNA and circ_0001971 siRNA by changing the dosage from 50 to 100 nM. Accordingly, as indicated in Fig. 6, the knockdown of hsa_circ_0001971 (Fig. 6A) dose-dependently suppressed the expression of mir-186 (Fig. 6B) while increasing the expression of SHP2 mRNA (Fig. 6C), while the knockdown of hsa_circ_0001874 (Fig. 6D) down-regulated the expression of miR-296 (Fig. 6E) and up-regulated the expression of PLK1 mRNA (Fig. 6F) in a dose-dependent manner.

Therefore, two signaling pathways, circ_0001971/miR-186/SHP2 and circ_0001874/miR-296-5p/PLK1, were established.

Moreover, it is noteworthy that, unlike SHP2 mRNA, the expression of PLK1 mRNA was significantly increased both by the overexpression of circ_0001971 and circ_0001874 (Fig. 4D). Similarly, the expression of PLK1 mRNA was evidently suppressed by the knockout of circ_0001971 or circ_0001874 (Fig. 5F), which indicated that the expression of PLK1 mRNA was not solely associated with the expression of its target miRNA miR-296-5p. Therefore, we suspected that the expression of PLK1 mRNA may also be associated with the expression of SHP2 mRNA.

### Synergistic effect of circ_0001971 and circ_0001874 on cell proliferation and apoptosis

**Figure 7:**
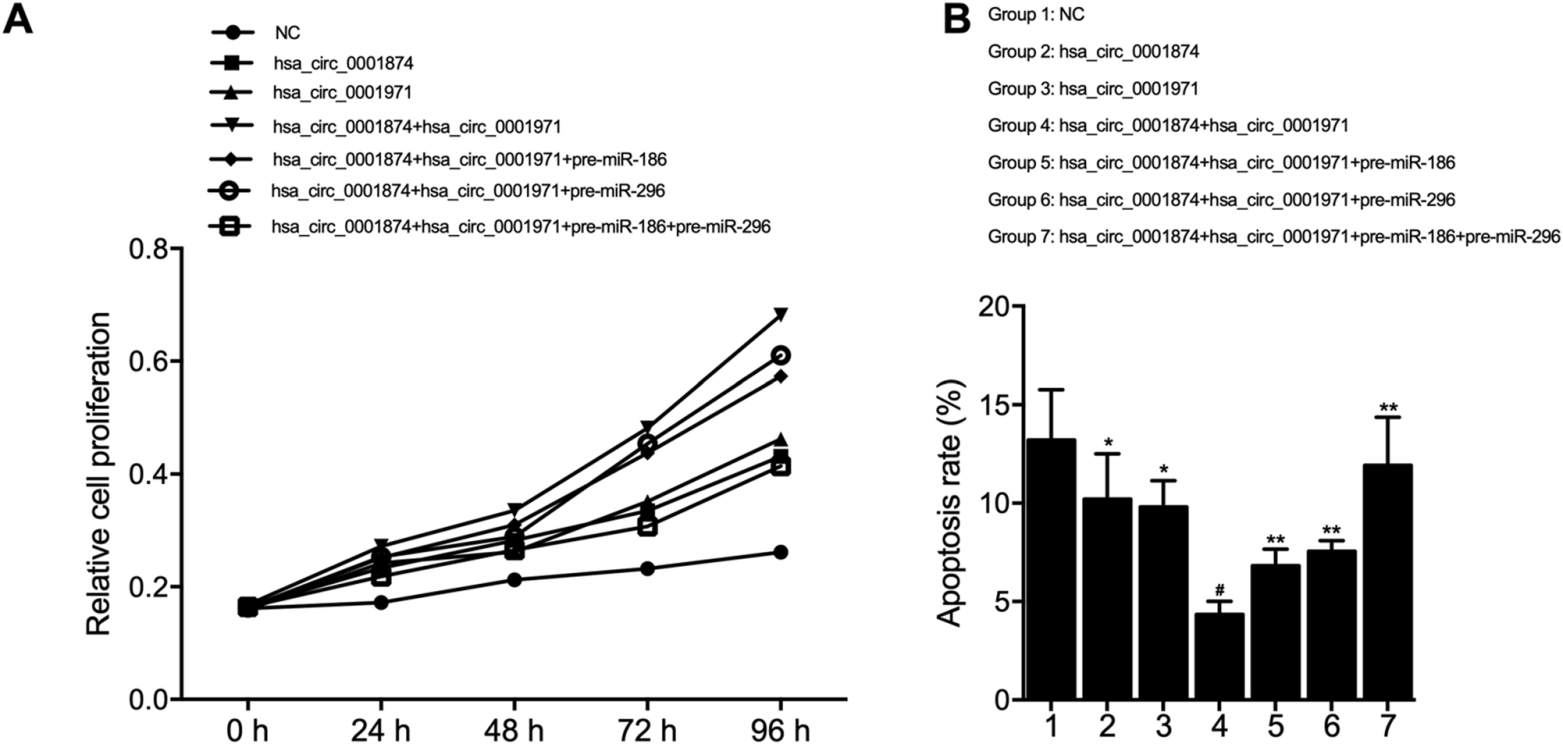
The overexpression of circ_0001971 and circ_0001874 synergistically promoted cell proliferation and suppressed cell apoptosis, while the overexpression of miR-186 and miR-296 obstructed the above effects. (**A**) Proliferation indicated by OD value at 570 nm was most increased in the hsa_circ_0001874 + hsa_circ_0001971 group. Although the proliferation was comparable between the hsa_circ_0001874 group and hsa_circ_0001971 group, the proliferation was the lowest in the NC group. Moreover, the up-regulation of miR-186 and/or miR-296 suppressed cell proliferation promoted by circRNA overexpression; (**B**) Apoptosis rate was most reduced in the hsa_circ_0001874 + hsa_circ_0001971 group. Although the apoptosis rate was comparable between the hsa_circ_0001874 group and hsa_circ_0001971 group, the apoptosis rate was the highest in the NC group. Meanwhile, the up-regulation of miR-186 and/or miR-296 increased apoptosis rate suppressed by circRNA overexpression (*P value < 0.05 vs. NC group; # P value < 0.05 vs. hsa_circ_0001874 group; **P value < 0.05 vs. hsa_circ_0001874 + hsa_circ_0001971 group).

MTT and FCM assays were performed to observe the synergistic effect of circ_0001971 and circ_0001874, and the effect of miR-186/miR-296 overexpression, on the proliferation and apoptosis of SCC-9 cells. As shown in Fig. 7A, the OD value of the negative control cells was the lowest, while the SCC-9 cells over-expressing both circ_0001971 and circ_0001874 showed the highest proliferation rate. Meanwhile, the transfection of miR-186 or miR-206 precursors suppressed circRNA-induced proliferation of SCC-9 cells, and the co-transfection of miR-186 and miR-206 precursors most significantly reduced circRNA-induced cell proliferation. On the contrary, the results in Fig. 7B showed that the apoptosis rate of SCC-9 cells was decreased after over-expressing circ_0001971 or circ_0001874. Compared with the NC group, circ_0001971 or/and circ_0001874 significantly suppressed cell apoptosis. The presence of miR-186 precursors and/or miR-206 precursors also increased the apoptosis of SCC-9 cells over-expressing circ_0001971 or circ_0001874.

### The level of miR-186 and miR-296-5p was lower in saliva of OSCC patients compared with that in OLK patients

**Figure 8:**
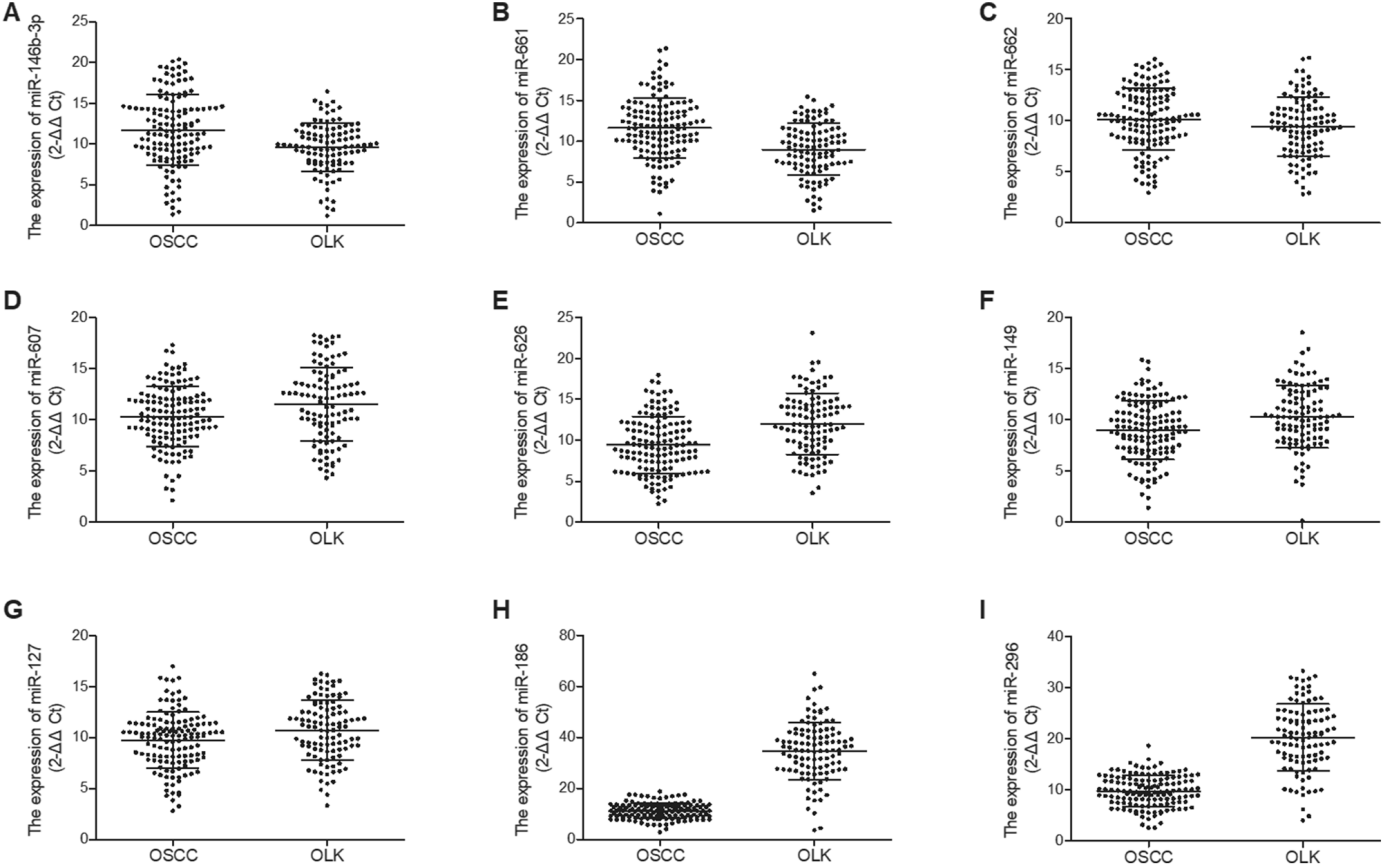
Among all candidate miRNAs, only the expression of miR-186 and miR-296-5p was reduced in saliva of OSCC patients compared with that in OLK patients. (**A**) The expression of miR-146b-3p was comparable in saliva of OSCC patients and OLK patients; (**B**) The expression of miR-661 was comparable in saliva of OSCC patients and OLK patients; (**C**) The expression of miR-662 was comparable in saliva of OSCC patients and OLK patients; (**D**) The expression of miR-607 was comparable in saliva of OSCC patients and OLK patients; (**E**) The expression of miR-626 was comparable in saliva of OSCC patients and OLK patients; (**F**) The expression of miR-149 was comparable in saliva of OSCC patients and OLK patients; (**G**) The expression of miR-127 was comparable in saliva of OSCC patients and OLK patients; (**H**) The expression of miR-186 was evidently lower in saliva of OSCC patients than that in OLK patients; (**I**) The expression of miR-296-5p was evidently lower in saliva of OSCC patients than that in OLK patients.

Two groups of patients were recruited in this study: A total of 135 OSCC patients were recruited into the OSCC group and a total of 105 oral leukoplakia (OLK) patients were recruited into the OLK group. The expression of several miRNAs was measured via RT-qPCR using saliva samples collected from the patients. As indicated by the results, no obvious difference was found between the two patient groups in terms of the expression of miRNAs including miR-146b-3p (Fig. 8A), miR-661 (Fig. 8B), miR-662 (Fig. 8C), miR-607 (Fig. 8D), miR-626 (Fig. 8E), miR-149 (Fig. 8F) and miR-127 (Fig. 8G). The relative expression of miR-186 (Fig. 8H) and miR-296-5p (Fig. 8I) was lower in the OSCC group compared with that in the OLK group.

### The level of miR-186 and miR-296-5p was up-regulated in saliva of post-operative OSCC patients

**Figure 9:**
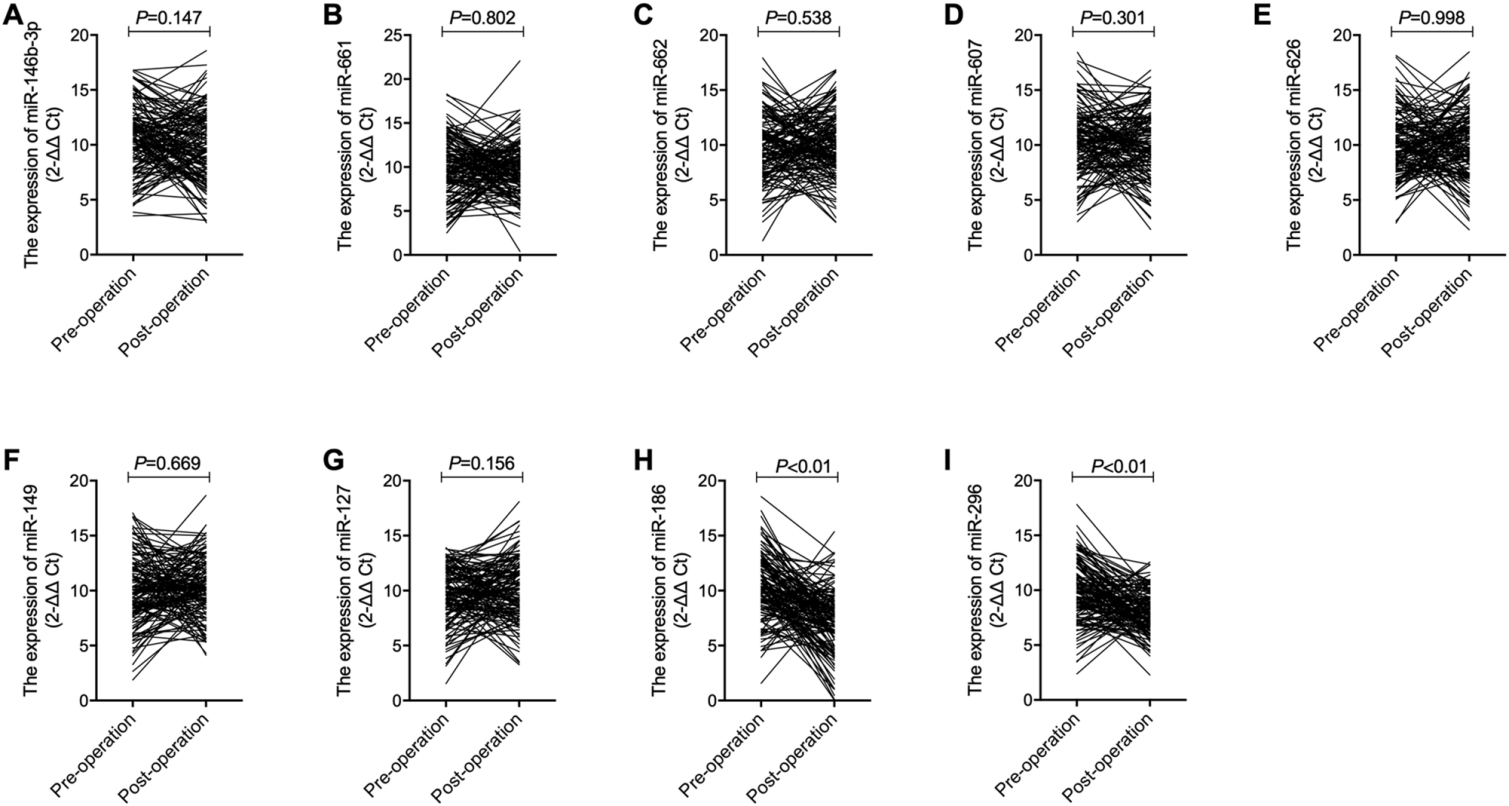
Among all candidate miRNAs, only the expression of miR-186 and miR-296-5p was up-regulated in saliva of post-operative OSCC patients. (**A**) The expression of miR-146b-3p was comparable in saliva of both pre-operative and post-operative OSCC patients; (**B**) The expression of miR-661 in was comparable in saliva of both pre-operative and post-operative OSCC patients; (**C**) The expression of miR-662 was comparable in saliva of both pre-operative and post-operative OSCC patients; (**D**) The expression of miR-607 was comparable in saliva of both pre-operative and post-operative OSCC patients; (**E**) The expression of miR-626 was comparable in saliva of both pre-operative and post-operative OSCC patients; (**F**) The expression of miR-149 was comparable in saliva of both pre-operative and post-operative OSCC patients; (**G**) The expression of miR-127 was comparable in saliva of both pre-operative and post-operative OSCC patients; (**H**) The expression of miR-186 was evidently up-regulated in saliva of both pre-operative and post-operative OSCC patients; (**I**) The expression of miR-296-5p was evidently up-regulated in saliva of both pre-operative and post-operative OSCC patients.

The expression of candidate miRNAs was also measured in saliva samples collected from the OSCC patients before and after operation. As shown in Fig. 8, the expression of miRNAs including miR-146b-3p (Fig. 9A), miR-661 (Fig. 9B), miR-662 (Fig. 9C), miR-607 (Fig. 9D), miR-626 (Fig. 9E), miR-149 (Fig. 9F) and miR-127 (Fig. 9G) was comparable in the pre-operative and post-operative OSCC groups. However, the expression of miR-186 (Fig. 9H) and miR-296-5p (Fig. 9I) was significantly down-regulated in saliva of post-operative OSCC patients.

## Discussion

In this study, we enrolled two groups of patients, including a total of 135 OSCC patients and a total of 105 OLK patients, to show that the relative expression of miR-186 and miR-296-5p was lower in the OSCC group compared with that in the OLK group. We also found that the expression of miR-186 and miR-296-5p was significantly down-regulated in saliva of OSCC patients after operation. Past studies have found differentially expressed circRNAs in the saliva collected from OSCC patients and healthy subjects, and hsa_circ_0001874 and hsa_circ_0001971 were pinpointed as potential markers in OSCC diagnosis^9^. In this study, we identified that the tumorigenic effect of hsa_circ_0001874 and hsa_circ_0001971 may be mediated by miR-186 and miR-296-5p. This was consistent with the previous reports about the roles of miR-186 and miR-296-5p in tumorigenesis.

A previous study has shown that miR-186 was downregulated in the tumor tissues of OSCC, while the over-expression of miR-186 could reduce cell proliferation. The investigation carried out by Ries et al. also suggested that the reduced blood level of miR-186 may be caused by the reduced miR-186 release from OSCC tissues^20^. Therefore, the blood level of miR-186 may be used as a biomarker for OSCC diagnosis^11^. Another study showed that miR-186 is downregulated in OSCC, and the expression of miR-186 in OSCC tissues is reversely associated with the expression of SHP2^11^. The Shp2 protein in OSCC is actually upregulated and linked to the stage of OSCC progression and the metastasis in lymph nodes, supporting the role of Shp2 as a gene that is related with tumorigenesis in OSCC^16^. The expression of miR-186 is substantially lower in the tumor tissues of OSCC, and miR-186 tends to inhibit cell growth while inducing apoptosis via the AKT and ERK signaling pathways through targeting SHP2^16^. It was also demonstrated that Shp2 plays a vital role in maintaining chromosomal stability^17^. In a previous study, microarrays were used to compare the profiles of circRNA expression in saliva of OSCC patients and healthy controls. The results indicated the possibility of using salivary hsa_circ_0001874 and hsa_circ_0001971 as biomarkers for OSCC diagnosis^9^. It was also shown that miR-296-5p and miR-129-5p expression was down-regulated in OLK-OSCC patients compared to that in the tissues of OLK patients, indicating that miR-296-5p and miR-129-5p may be associated with inhibition of tumor growth^21^. Moreover, a previous research explored whether miR-296-5p reduced the viability of NSCLC cells by targeting the expression of PLK1. By utilizing luciferase assays, it was effectively shown that miR-296-5p can directly target the expression of PLK1 mRNA through binding to its 3′-UTR^19^. It was also revealed that the expression of PLK1 was needed for the transition of OSCC cells from the G2 phase to the M phase, likely by means of CCNB1 expression and phosphorylation^22^. It was also shown that ER maleate worked as an anti-cancer agent in OSCC treatment by inducing the apoptosis of OSCC cells, inhibiting the migration and invasion of OSCC cells, and inhibiting the division of OSCC cells^23^. In this study, SHP2 was confirmed as a target gene of miR-186 and PLK1 was confirmed as a direct target gene of miR-296. To establish the according molecular mechanisms, the interactions between circRNAs including circ_0001971 and circ_0001847 and miRNAs including miR-186 and miR-296-5 were also explored.

CircRNAs have been revealed to function as miRNA sponges, and regulate their transcription or splicing, since their circular structure is stable and their sequences are conserved. For example, ciRS-7/CDR1as has been reported as molecules which contains more than 70 binding sites of miR-7, and it can adsorb miR-7 to reduce its activity^24^. The overexpression of CDR1as in zebrafish embryos participated in the development of midbrain via sponging miR-7^25^. Moreover, the binding between CDR1as and miR-7 also inhibited the biological function of miR-7, leading to a significant regulatory effect in cancer-associated pathways^26^. Similar relationships were also found in other circRNAs and miRNAs. For example, circ-ITCH was found to function as a sponge of miR-7, miR-17 and miR-124, leading to the inhibition of cell proliferation and tumor growth in colorectal cancer and esophageal squamous cell carcinoma respectively^27^. Overall, circRNA functions as a sponge of miRNA to regulate its level and therefore the expression level of the direct targets of the miRNA but the reverse regulation of circRNA by miRNA was not that much possibly due to the stable nature of a circular structure. miR-671 and miR-7 have been reported to regulate expression of circRNA-CDR1as, and this make the regulation between the circRNA and miRNA bidirectional^28^. In this study, we found that the over expression of miR-296-5p and miR-186 could respectively bind to circ_0001847 and circ_0001971 and substantially down-regulated the level of these circRNAs, which indicated that these circRNAs and miRNAs sponged each other. Since SHP2 mRNA and PLK1 mRNA were respectively targeted by miR-186 and miR-296-5p, we therefore established two signaling pathways of circ_0001971/miR-186/SHP2 and circ_0001874/miR-296-5p/PLK1 which regulated the oncogenic effects of hsa_circ_0001971 and hsa_circ_0001874 in the development of OSCC. This verified our hypothesis that the effect of miR-186/miR-296-5p might be mediated by its interaction with ceRNAs such as circ_0001971 and circ_0001874 to affect the expression and function of SHP2 and PLK1.

Interestingly, the overexpression (Fig. 4) or silencing (Fig. 5) of hsa_circ_0001971 upregulated or downregulated PLK1 respectively without affecting miR-296-5p expression, which indicated the cross-talk between the two signaling pathways. In line with this, it has been found that SHP2 is a necessary activator for the oncogenic effect of PLK1^29^, and hsa_circ_0001971 dysregulation may affect both SHP2 and PLK1, but hsa_circ_0001874 only affects PLK1. Furthermore, we also found that, as endogenous competing RNA of the corresponding RNA, the relative level of miR-295 and miR-186 was lower in the saliva of OSCC group compared with OLK group, while being higher in the saliva of post-operation samples compared with the pre-operation samples, indicating that those two miRNAs could be novel biomarkers for the development of OSCC or prognostic biomarkers for those patients who received surgical intervention.

However, the results obtained from our study were limited. First of all, the sample size was not large enough to draw a decisive conclusion regarding the role of circRNAs as biomarkers in OSCC development. Secondly, we did not collect the tumor samples from the patients. Thirdly, no clinical trials were performed to validate the hypothesis of the suppressive effect of circRNA down-regulation in the controlling of OSCC risk.

## Conclusion

In conclusion, the finding of this study demonstrated that hsa_circ_0001971 and hsa_circ_0001874 were differentially expressed in OSCC and may be used as predictive biomarkers for the development of OSCC. Furthermore, the oncogenic effects of hsa_circ_0001971 and hsa_circ_0001874 in the development of OSCC might be, at least partially, mediated by their downstream signaling pathways hsa_circ_0001971/microRNA-186/SHP2 and hsa_circ_0001874/microRNA-296/PLK1.

## Data Availability

The data that support the findings of this study are available from the corresponding author upon reasonable request.
